# Arbuscular mycorrhizal associations persist in flooded sediment, with distinct communities in soil-borne and sediment-borne roots of *Phragmites australis*

**DOI:** 10.3389/ffunb.2026.1815819

**Published:** 2026-07-15

**Authors:** Joao Wendrich Teixeira, Justin D. Stewart, Nicola T. Case, Bethan F. Manley, E. Toby Kiers, Vasilis Kokkoris

**Affiliations:** 1 Amsterdam Institute for Life and Environment (A-LIFE), Section Systems Ecology, Vrije Universiteit Amsterdam, Amsterdam, Netherlands; 2 Society for the Protection of Underground Networks (SPUN), Wilmington, DE, United States; 3 Amsterdam Institute for Life and Environment (A-LIFE), Section Ecology and Evolution, Vrije Universiteit Amsterdam, Amsterdam, Netherlands

**Keywords:** colonisation, common reed, diversity, flooding, mycorrhizal fungi, wetland

## Abstract

Arbuscular mycorrhizal (AM) fungi form symbioses with most terrestrial plant species, and likely facilitated the transition of plants from aquatic to terrestrial environments. Surprisingly, the plant-AM symbiosis remains understudied in aquatic and water-saturated habitats. Here, we assessed AM fungal colonisation and community composition in the roots of a widespread wetland grass, *Phragmites australis* (common reed), in the Netherlands. Samples were collected in dry soil (soil-borne) and in flooded sediments (sediment-borne) up to 1 meter below water. We quantified AM fungal colonisation in both soil-borne and sediment-borne roots, with approximately 80% of samples showing AM fungal colonisation across both root habitats. Soil-borne roots showed higher colonisation frequency (mean 50% ± 30 SD) than flooded sediment-borne roots (mean 39% ± 25 SD). We then used amplicon sequencing (18S rDNA) to explore the community composition of roots, recovering an average of ~20 AM fungal taxa (ASVs richness estimates) from soil-borne roots and ~9 ASVs from flooded sediment-borne roots. Soil-borne and sediment-borne roots harboured different AM fungal communities, with varied genera present between the two. Together, our results provide an initial baseline for understanding AM fungal diversity associated with *P. australis* and suggest that differences in community composition between soil-borne and sediment-borne roots may reflect flooding-driven environmental filtering.

## 1 Introduction

Arbuscular mycorrhizal (AM) fungi form symbioses with the roots of >70% of terrestrial plant species (Brundrett and Tedersoo, 2018). AM fungi supply their host plants with soil nutrients and water in exchange for photosynthetically fixed carbon through a network of hyphae that extend both within (intraradical), and outside plant roots (extraradical). Extraradical hyphae proliferate in the surrounding soil or sediment, where they acquire nutrients and water, while intraradical hyphae colonise root cortical cells and facilitate nutrient exchange with the plant host (Smith and Read, 2008; Hawkins et al., 2023). At local spatial scales, AM fungi influence plant health and soil structure (Leifheit et al., 2014). Globally, AM fungi are responsible for substantial carbon drawdown, with an estimated ~1.0 gigaton of carbon allocated to AM fungal mycelium annually (Hawkins et al., 2023).

The mycorrhizal symbiosis is thought to have facilitated plant transition from aquatic to terrestrial environments more than 400 million years ago (Brundrett, 2002; Humphreys et al., 2010). Yet, despite the potential aquatic origin of this symbiosis, AM fungal research has overwhelmingly focused on terrestrial ecosystems. Aquatic plants account for only ~6% of plant species represented in the largest AM fungal environmental DNA database, with field samples restricted to just two aquatic biomes within a small portion of the world’s ecoregions (Větrovský et al., 2023; Stewart et al., 2024; Stewart et al., 2025). As a result, the prevalence and diversity of AM fungal symbioses in aquatic environments remain poorly characterised.

This knowledge gap is particularly problematic considering the ecological importance of wetland ecosystems. Wetlands cover approximately 8% of Earth’s surface, functioning as biodiversity hotspots and major carbon sinks (Berde et al., 2022). At the same time, wetlands are among the most threatened ecosystems globally with approximately 87% of wetlands degraded or in decline, largely due to agricultural expansion and urbanisation (Walpole and Davidson, 2018). Wetland degradation and loss may threaten AM fungal diversity before its composition, functions, and ecological importance have been fully characterized.

Understanding AM fungal ecology in wetlands is challenging because flooded environments impose strong abiotic constraints that may limit the ability of AM fungi to survive, colonise plant roots, and establish communities, relative to terrestrial systems. Reduced oxygen availability, altered nutrient diffusion, and sediment instability may influence fungal growth and nutrient acquisition strategies (Stevens et al., 2011; Xu et al., 2021). In addition, variation in root architecture and function among wetland plants may further shape AM fungal colonisation by altering oxygen availability, nutrient acquisition strategies, and root–soil contact, thereby generating heterogeneous microenvironments for fungal establishment (Dreyer et al., 2014; Wen et al., 2019; Bergmann et al., 2020; Xu et al., 2021).

Despite these constraints, some wetland plant species are known to associate with AM fungi (Hu et al., 2020; Nielsen et al., 2004; Ramírez-Viga et al., 2018; Søndergaard and Laegaard, 1977; Wen et al., 2019; Xu et al., 2016; Xu et al., 2021; Calheiros et al., 2019; Radhika and Rodrigues, 2007) (**Supplementary Table 1**), and recent global analyses predict wetlands to be centres of AM fungal diversity and endemism (Van Nuland et al., 2025). Similar to their functions in terrestrial ecosystems, associations among wetland plants and AM fungi have been shown to enhance tolerance to nutrient limitation, salinity, and heavy metal stress (Wang et al., 2010; Fester, 2013; Ramírez-Viga et al., 2018; Gao et al., 2020; Hu et al., 2021). However, it remains unclear to what extent AM fungal associations persist under permanently flooded conditions and whether flooding acts as an environmental filter structuring AM fungal communities.

*Phragmites australis* (common reed) provides a useful system to address these questions due to its pronounced root plasticity allowing widespread occurrence across flooded and non-flooded wetland habitats. This cosmopolitan reed species is widely used for pollutant removal (DeBusk et al., 1990; Garbett, 2005; Headley et al., 2005), habitat provision (Hardman et al., 2012; Vermaat et al., 2016; Knoblauch and Gander, 2020), and in some regions, for energy production from biomass and as construction material (Važić et al., 2015; Caponetto et al., 2023). Importantly, *P. australis* occurs continuously across terrestrial and aquatic environments without clear habitat separation, exposing associated AM fungi to strong hydrological gradients and contrasting abiotic conditions within the same host species. This makes *P. australis* a useful natural system for examining how flooding influences AM fungal colonisation and community assembly while minimizing differences associated with host identity.

Here, we assessed AM fungal colonisation and diversity in *P. australis* roots across two Dutch wetland nature reserves, sampling reed roots from two distinct environments: dry soil and flooded sediment. By comparing roots exposed to contrasting hydrological conditions within the same host species, we aimed to evaluate whether flooding alters AM fungal colonisation patterns and community assembly. Specifically, we asked: (1) To what extent do AM fungi colonise soil-borne and sediment-borne reed roots, and does colonisation vary among the two environments? (2) What levels of AM fungal richness (i.e. diversity) are associated with reed roots? and (3) Does AM fungal community composition vary among these environments? Given the abiotic constraints associated with flooding, we predicted that sediment-borne roots would exhibit lower AM fungal colonisation and distinct AM fungal community composition relative to soil-borne roots.

## 2 Materials and methods

### 2.1 Sampling locations

We sampled *P. australis* roots from two long-managed reed harvesting fields located within protected wetland reserves in Friesland, the Netherlands. Specifically, we collected soil-borne (terrestrial) and flooded sediment-borne (permanently submerged) roots of *P. australis* from two ~100-year-old reed harvesting fields found within these protected areas - Rottige Meenthe: 52.84755448, 5.8967188 and Brandemeer: 52.874261, 5.879581. Both sites have similarly mean annual temperatures (13 °C) and mean annual precipitation (840 mm) (Royal Netherlands Meteorological Institute (KNMI), 2020). Sampling across two wetland reserves allowed us to evaluate whether patterns in AM fungal colonisation and community composition were reproducible across independent sites with similar wetland characteristics, while reducing the likelihood that observed differences were driven by site-specific environmental variation alone. Although these are designated nature reserves, traditional seasonal harvesting of aboveground reed biomass is still practiced as part of long-term wetland management within the area.

### 2.2 Sample collection

At both sites, root samples were divided into soil-borne and sediment-borne groups. Soil-borne roots were collected from soils at 15–30 cm depth. Sediment-borne roots were collected from permanently flooded sediments, with the sediment surface overlain by 0.5–1.0 m of water. In total we collected 80 samples: 20 soil-borne root samples and 20 sediment-borne samples per location (Rottige Meenthe and Brandemeer). After collection, roots were gently cleaned of adhering sediment and subdivided for microscopic assessment of mycorrhizal colonisation and for DNA-based characterisation of AM fungal communities. Washed root subsamples were stored in 70% ethanol at 4 °C (less than 3 days) until further processing.

### 2.3 Assessment of AM fungal colonisation

Root samples were cleared and stained using the ink-and-vinegar method (Vierheilig et al., 1998). For each sample (n=20 per category), 15 2cm long stained root fragments were randomly selected and mounted on microscopy slides and examined using light microscopy (ZEISS Axio Imager M2). AM fungal colonisation was assessed by scoring the presence of characteristic intraradical structures, including hyphae, vesicles, and arbuscules, following established methods (Trouvelot and Gianinazzi, 1986; Kokkoris et al., 2019). Briefly, the intensity of colonisation per root fragment was scored on a scale of 0-5, while arbuscule and vesicle abundance were scored per root fragment on a scale of 0-3 (**Supplementary Figure 1**, **Supplementary Methods**). The intensity of colonisation (M%), arbuscule abundance (A%), and vesicle abundance (V%) was then calculated for each root system, which corresponds to all 15 fragments assessed per sample (**Supplementary Methods**). The frequency of colonisation (F%) was determined as the percentage of the root system that contain AM fungal structures regardless of type or quantity (**Supplementary Methods**).

### 2.4 DNA extraction and sequencing

For each root sample, root material was frozen using liquid nitrogen and ground using a porcelain mortar and pestle. DNA was extracted from ground root material using a Qiagen DNeasy PowerSoil Pro Kit (Helden, Germany), following the manufacturer’s protocol. Negative controls, including an empty-grinder control processed with liquid nitrogen and an extraction kit control, were included to monitor potential contamination during sample processing and DNA extraction. These negative controls were not sequenced because they did not show amplification. A positive control consisting of *in vitro*-derived *Rhizophagus irregularis* DNA was included during PCR to confirm successful amplification and the expected amplicon size.

Metabarcoding was used to quantify AM fungal richness and community composition. Each of the 4 sample groups (soil-borne roots from Brandemeer, sediment-borne roots from Brandemeer, soil-borne roots from Rottige Meenthe and sediment-borne roots from Rottige Meenthe) were each pooled in 3 composite samples, with a total of 12 DNA composite samples that were used for DNA sequencing. As previously (Stewart et al., 2026), the SSU region was amplified using an AM fungi-specific primer set, WANDA (5’ – CAG CCG CGG TAA TTC CAG CT – 3’)/AML2 (5’ – GAA CCC AAA CAC TTT GGT TTCC – 3’) (Lee et al., 2008; Dumbrell et al., 2011). Amplicons were cleaned up with 1x AMPure beads, indexed with Illumina UD Indexes following the manufacturer’s protocol, pooled at equimolar concentration, and sequenced using an Illumina NextSeq 2000 (2 x 300 paired-end chemistry) at Scripps Research, USA.

SSU raw reads were quality filtered and processed using LotuS2 V2.34 (Özkurt et al., 2022) into amplicon sequence variants (ASVs) using DADA2. To ensure that only AM fungi ASVs were retained, an edited version of SILVA v138.2 (Quast et al., 2013) with Glomeromycota sequences removed, was used to identify and remove non-AM fungal ASVs. Taxonomy of the retained ASVs was assigned using MaarjAM virtual taxa (VT) with adjusted nomenclature (Öpik et al., 2010), using 98% coverage and 97% similarity thresholds. To assign mycorrhizal status, AM fungi were identified by sub-setting ASVs with taxonomic assignment to the classes of Glomeromycetes, Archaeosporomycetes, and Paraglomeromycetes.

Putative contaminants were filtered out based on the presence and abundance of ASVs in negative control samples using the *decontam* package in R (Davis et al., 2018), using a cutoff threshold of 0.1. Samples with less than 500 reads were removed and ASVs and OTUs with less than 0.01% of the total read count per sample were removed to account for putative index switches. The metabarcoding dataset was visualised using *phyloseq* (McMurdie and Holmes, 2011).

### 2.5 Statistical analyses

Root colonisation metrics were analysed using the Kruskal-Wallis test, as data showed non-normally distributed residuals when a parametric ANOVA was used. Next, the Dunn test with a *post-hoc* Bonferroni correction for multiple testing was performed to understand how colonisation scores varied between root habitat (soil-borne *vs* sediment-borne) and sampling location (Rottige Meenthe *vs* Brandemeer). All tests were conducted in R studio (version 4.3.2) (Posit Team, 2025).

Richness (Chao1 index) and diversity (Shannon index) were calculated using the iNEXT package on ASVs (McMurdie and Holmes, 2011; Oksanen et al., 2025) and an ANOVA was used to test for differences between root habitat and sampling location. To determine community composition, ASV counts were converted to relative abundances and Bray-Curtis dissimilarities were calculated. A permutational ANOVA (PERMANOVA) was then performed with site and root type as fixed effects using 999 permutations. Tests for homogeneity of dispersion (n=999) indicated no differences by location (p = 0.157) or root habitat (p = 0.901), confirming that the assumptions of PERMANOVA were met. Community patterns were visualised using principal coordinates analysis (PcoA).

## 3 Results

### 3.1 Root colonisation

**Figure 1:**
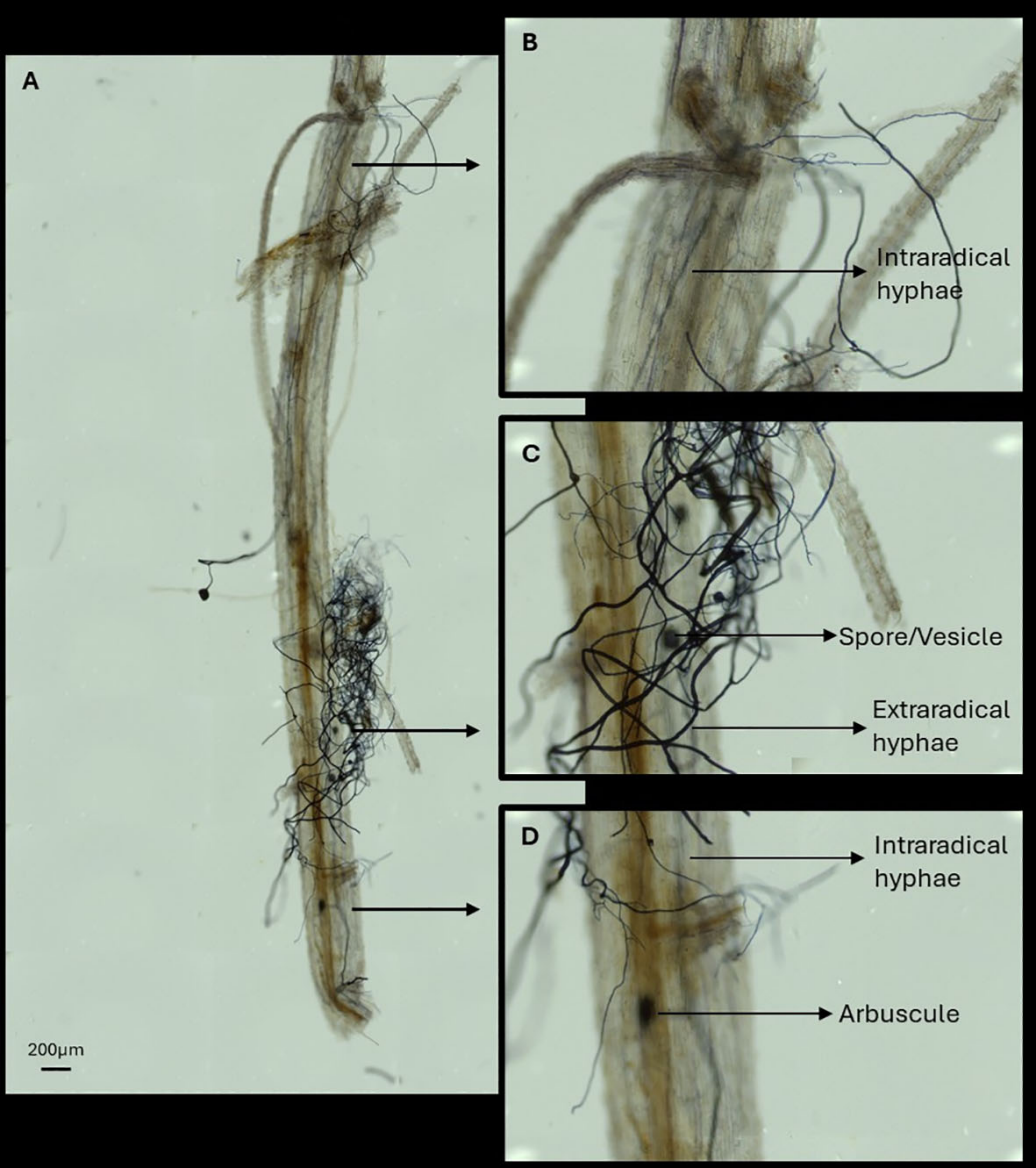
Arbuscular mycorrhizal (AM) fungal colonization of *Phragmites australis*. roots. **(A)** Stitched light microscopy image compiled from 32 individual panels, showing AM fungal colonization along a reed root fragment collected from flooded sediment. Arrows indicate regions shown at higher magnification in panels **(B–D)**. **(B–D)** Enlarged microscopy panels highlighting representative AM fungal structures observed within and around reed roots, including intraradical hyphae, extraradical hyphae, spores, vesicles, and arbuscules. Scale bar in panel A = 200 µm. Images were obtained using a ZEISS Axio Imager M2.

**Figure 2:**
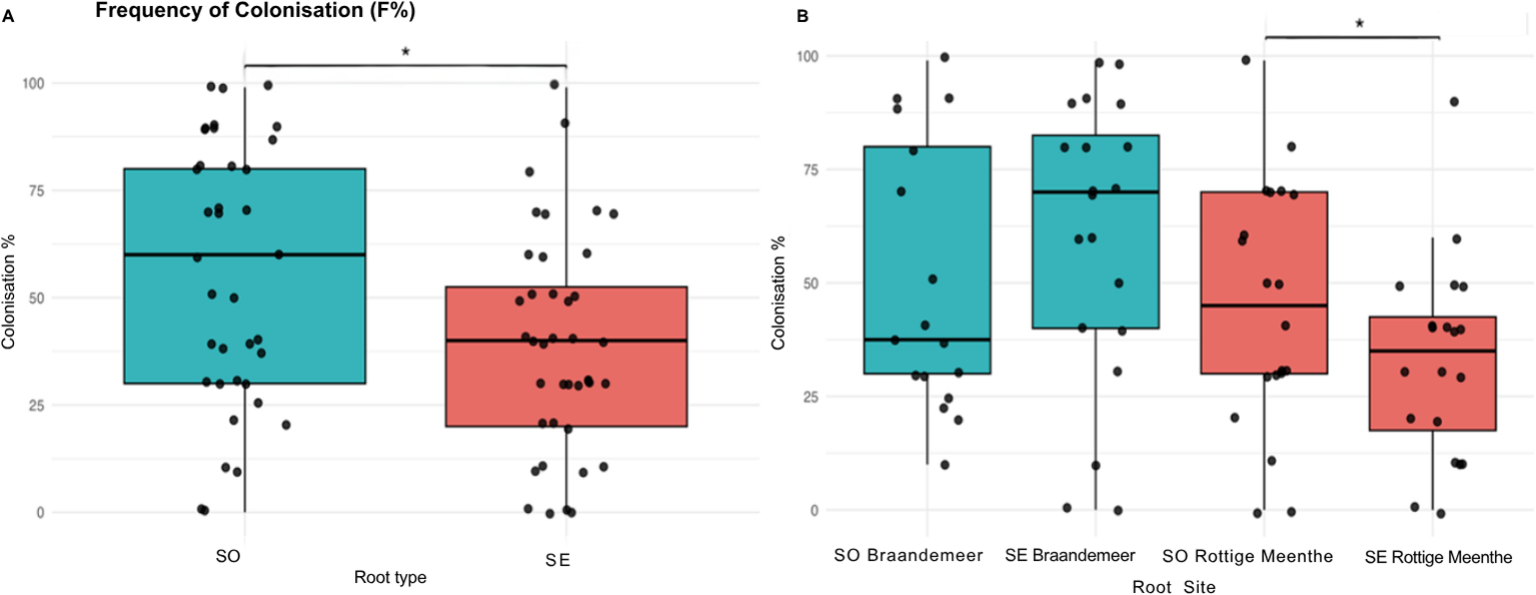
AM fungal colonisation between reed root habitats and sampling sites. AM fungal colonisation in *Phragmites australis* roots collected from two habitat types and two wetland sites. **(A)** Frequency of colonisation, expressed as the percentage of the root system showing AM fungal structures per sample (F%), compared between soil-borne roots (SO) and flooded sediment-borne roots (SE). **(B)** Frequency of colonisation shown separately for each root habitat within the two sampling sites, Braandemeer and Rottige Meenthe. Boxplots show the median as the central bold line, the interquartile range as the lower and upper hinges, and whiskers extending to the 5th and 95th percentiles. Individual points represent biological samples. Colours indicate root habitat, with soil-borne roots shown in blue and sediment-borne roots in red. Differences between groups were tested using a Kruskal–Wallis test. Asterisks indicate significant differences between groups (*p* < 0.05).

We detected AM fungi in roots collected from both habitats, including in roots growing up to one meter below the water surface (**Figure 1**). When examining the frequency of colonisation (F%) of reed roots across root habitat, we found soil-borne roots to have significantly higher presence of AM fungal structures (mean 50% +/- 30 SD) than sediment-borne roots (mean 39 +/- 25 SD) (p = 0.0096, Kruskal-Wallis test; **Figure 2A**). When examining the frequency of AM colonisation in Rottige Meenthe, we found that soil-borne roots showed significantly more colonisation (mean 60.4+/- 31.4 SD) than sediment-borne roots (mean 33 +/- 22 SD) (p= 0.01, Kruskal-Wallis test; **Figure 2B**). In Brandemeer, there were no significant differences between the soil-borne (mean 49.9 +/- 29.4 SD) and sediment-borne roots (mean 45 +/- 27.3 SD) (p = 1, Kruskal-Wallis test).

Across root habitat (soil-borne *vs* sediment-borne roots) and across sampling location (Braandemeer *vs* Rottige Meenthe), the intensity of colonisation (M%), arbuscule abundance (A%), and vesicle abundance (V%) at the level of the root system did not show any significant differences (p > 0.05, Kruskal-Wallis test; **Supplementary Figures 2**, **S3**, **S4**, respectively).

### 3.2 AM fungal diversity and community composition

**Figure 3:**
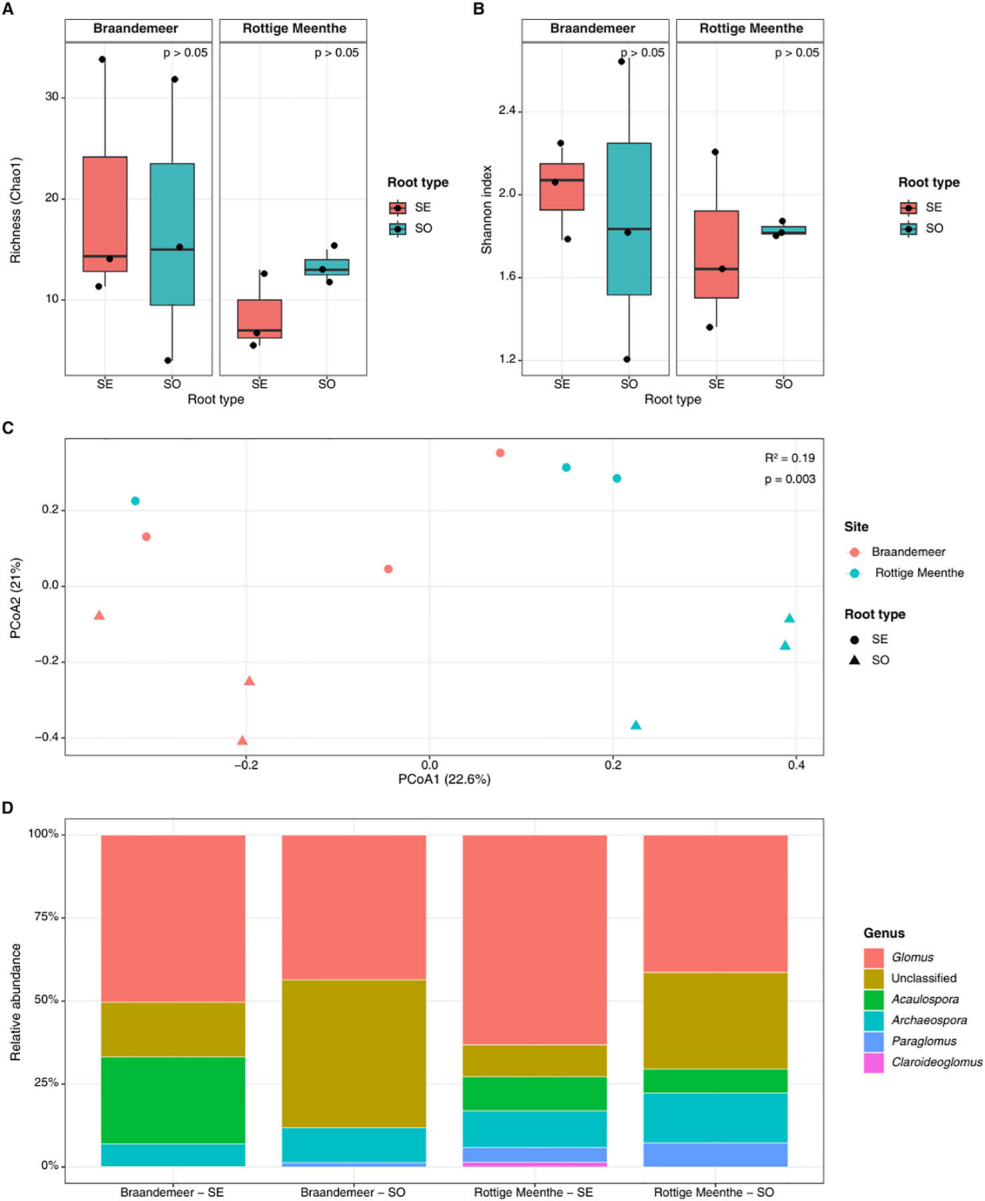
AM fungal diversity and community composition across reed root habitats and wetland sites. AM fungal communities associated with *Phragmites australis* roots collected from soil-borne SO and flooded sediment-borne SE habitats at Braandemeer and Rottige Meenthe. **(A)** AM fungal richness, estimated as Chao1 richness, across root habitats within each sampling site. **(B)** AM fungal alpha diversity, estimated using the Shannon index, across root habitats within each sampling site. For panels A and B, boxplots show the median as the central bold line, the interquartile range as the lower and upper hinges, and whiskers extending to the range of the data shown; individual points represent biological samples. **(C)** Principal coordinates analysis showing differences in AM fungal community composition among samples. Colours indicate sampling site and point shapes indicate root habitat. The percentage of variation explained by each axis is shown in parentheses. **(D)** Relative abundance of AM fungal genera detected across each site–habitat combination.

We detected 289,534 reads across 43 ASVs. Richness (Chao1 index) in sediment-borne roots at Braandemeer was 19.9 ± 12.3 SD ASVs, while Rottige Meenthe showed 8.5 ± 4.0 SD ASVs richness (**Figure 3A**). For soil-borne roots, we found a mean Chao1 values of 17.0 ± 14.1 SD ASVs at Braandemeer and 13.3 ± 1.5 SD ASVs at Rottige Meenthe (**Figure 3A**). Shannon diversity values were lower overall, where sediment-borne roots at Braandemeer had a diversity of 2.03 ± 0.23 SD, while at Rottige Meenthe this was 1.74 ± 0.43 SD (**Figure 3B**). Soil-borne roots showed mean Shannon values of and 1.90 ± 0.73 SD at Braandemeer and 1.83 ± 0.04 SD at Rottige Meenthe (**Figure 3B**). Neither root habitat nor site location influenced richness or Shannon diversity (ANOVA, p > 0.05).

AM fungal community composition varied significantly by root type (PERMANOVA, R² = 0.19, p = 0.003), while differences between sampling sites were marginally insignificant (p = 0.063; **Figure 3C**). Group dispersion did not significantly differ by site (permutational test, p = 0.157) or root type (p = 0.901), indicating that group variances were similar across site and root type. The PERMANOVA signal is not driven by dispersion, so differences reflect real shifts in community composition. We determined AM fungal community composition at the genus level (**Figure 3D**; **Supplementary Figure 5**). For sediment-borne roots in Brandemeer, *Glomus* were the most abundant (50.41%), followed by *Acaulospora* (26.23%) and *Archaeospora* (6.97%), with 16.39% of unclassified taxa. Notably, *Claroideoglomus* and *Paraglomus* were absent. In Rottige Meenthe, *Glomus* also dominated the sediment-borne roots (63.24%), followed by *Archaeospora* (11.02%), *Acaulospora* (10.29%), *Paraglomus* (4.41%) and *Claroideoglomus* (1.47%), with 9.56% unclassified taxa. For soil-borne roots in Brandemeer, the AM fungal community was composed largely of unclassified taxa (44.53%) and *Glomus* (43.61%) followed by *Archaeospora* (11.03%) and *Paraglomus* (1.28%), while *Acaulospora* and *Claroideoglomus* were absent. For soil-borne roots in Rottige Meenthe, *Glomus* was the dominant genus (41.36%), followed by unclassified taxa (29.09%), *Archaeospora* (15%), *Acaulospora* (7.27%) and *Paraglomus* (7.27%).

## 4 Discussion

Our aim was to evaluate the AM fungal colonisation and diversity in *P. australis* roots from two Dutch wetland sites, comparing sediment-borne and soil-borne reed roots. AM fungi were detected in roots from both habitats, demonstrating that *P. australis* can maintain mycorrhizal associations under permanently flooded conditions, reaching up to one meter underwater. However, the frequency of AM fungal colonisation was significantly higher in roots from dry soil than in those from flooded sediment. Although AM fungal communities showed similar levels of richness and diversity across root habitats (soil- *vs* sediment-borne), we found that community composition shifted significantly, reflecting habitat-driven changes. Together, these results show that fungal colonisation and the community composition vary with environmental conditions within wetland habitats.

The differences in the frequency of AM fungal colonization and community composition between soil-borne and sediment-borne roots are likely driven by a combination of abiotic constraints imposed by flooded conditions and biotic filtering linked to AM fungal functional traits. In terrestrial systems, AM fungi typically forage for nutrients such as phosphorus by extending their extraradical hyphal networks into the soil matrix, thereby increasing root nutrient acquisition beyond the depletion zone around the root. In contrast, freely dissolved nutrients in aquatic environments diffuse far more rapidly than in porous soils, potentially reducing the relative benefit of long-distance hyphal foraging and favouring fungi with shorter hyphal networks or more intraradical allocation (Stevens et al., 2011). For example, phosphorus diffusion coefficients in water can be orders of magnitude higher than in soils (Barraclough and Tinker, 2006; Bachmeier and Rollan, 2014), facilitating nutrient access without extensive proliferation of extraradical AM fungal hyphae. The increased availability of dissolved nutrients in water may also reduce plant dependency on AM fungi, resulting in decreased overall colonisation. Furthermore, under flooded conditions, oxygen availability also likely limits colonisation, as AM fungi are aerobic organisms (Andersen and Andersen, 2006; Albuquerque et al., 2020; Xu et al., 2021). While the rhizomes of reed plants may alleviate hypoxic pressure by transporting oxygen from shoots down to roots, effectively functioning as “air pipes” (Zhang et al., 2014; Albuquerque et al., 2020), oxygen availability may still impose limitations on AM fungal growth, resulting in lower colonization values overall.

Disturbance regimes and fungal life history traits might also shape AM fungal diversity and colonisation patterns in aquatic habitats. Some AM fungi, such as the model species *Rhizophagus irregularis*, can extend their extraradical hyphae, effectively take up phosphorus, and complete their life cycle via spore production in flooded conditions (Maldonado-Mendoza et al., 2001). However, extraradical hyphae are generally more vulnerable to water turbulence, which can physically disrupt hyphal networks (Bago and Cano, 2005). This may therefore select for taxa that allocate more into intraradical rather than extraradical growth. This dynamic may contribute to filtering effects on the AM fungal community: taxa that allocate more biomass to intraradical structures could be favoured in flooded sediments, while those with more extensive extraradical hyphae persist in drier soil environments. Indeed, AM fungal colonisation strategies differ markedly among taxa, reflecting trade-offs between intraradical proliferation and exploration of the soil matrix (Hart and Reader, 2002; Klironomos and Hart, 2002; Kokkoris, 2026). The observed variation in AM fungal colonization may be linked to differences in the AM fungal communities colonizing soil-borne and sediment-borne roots. In our dataset, root environment did not significantly affect ASV richness or Shannon diversity, suggesting that soil-borne and sediment-borne roots were colonized by a comparable number and evenness of AM fungal sequence variants. However, AM fungal community composition differed significantly between roots growing in dry soil and those growing in flooded sediment. This compositional shift supports the interpretation that flooded sediment conditions may act as an environmental filter, selecting for distinct AM fungal communities capable of colonizing roots under these conditions.

Specifically, we found that roots from flooded sediment were dominated by *Glomus* sp., which is in line with a previous study that found that *Glomus* sp. were the most common AM fungal taxon in flooded reeds (Chen et al., 2024). *Glomus* sp. are known to produce a higher proportion of hyphae within roots (intraradical hyphae) as opposed to extraradical hyphae, which may be beneficial in flooded conditions (Hart and Reader, 2002). While the same study by Chen et al. (2024), found that reed roots isolated from dry soils were dominated by *Paraglomus* sp., here we found that next to a large portion of unclassified taxa, *Glomus* sp. dominated roots isolated from dry soil. The observed discrepancy could be due to differences in geographical location as AM fungi exhibit highly variable geographic distributions, shaped by local climate, land use, and nutrient conditions (Stürmer et al., 2018; Van Nuland et al., 2025). Importantly, this discrepancy highlights the need to increase sampling of AM fungi in wetlands to better understand their community dynamics.

There are limitations to our study which are important to recognize. First, relatively few aquatic plants have been sampled for AM fungi (**Supplementary Table 1**), leaving our understanding of the global prevalence of AM fungal symbiosis with aquatic plants largely incomplete (Větrovský et al., 2023; Stewart et al., 2025). While our study of aquatic AM fungal communities in wetlands helps fill this gap, the work is limited to samples from only one geographical region and wetland reserve. It is also only focuses on a single host species. This means that other host and locations could provide comparably different results. Second, available data on fungal traits are almost entirely derived from terrestrial AM fungi, which brings uncertainty when attributing fungal traits to identified taxa in aquatic systems (Chaudhary et al., 2025; Oyarte Galvez et al., 2025). AM fungal traits significantly influence community composition and assembly in terrestrial ecosystems (Chagnon et al., 2013; López-García et al., 2014), but without comparable trait measurements from aquatic systems, we cannot assess drivers of AM fungal community assembly.

Our findings expand the view of AM fungal ecology beyond predominantly terrestrial systems by showing that reed roots in flooded sediments can harbour diverse AM fungal communities. Rather than representing a simple reduction of the terrestrial symbiosis, sediment-borne roots supported distinct community assemblages, suggesting that aquatic and water-saturated habitats may select for particular AM fungal taxa. This highlights wetlands as important, but still poorly characterized habitats for AM fungal diversity. Broader sampling across wetland plant species, hydrological gradients, and geographic regions will now be needed to determine how common these associations are, which fungal lineages are most consistently linked to flooded environments, and whether aquatic AM fungal communities follow ecological patterns distinct from those observed in soils.

## Data Availability

The original contributions presented in the study are publicly available. All code and data are available in our GitHub repository (https://github.com/joWeTe/Reed_AMF_project. DNA sequences are deposited in NCBI under accession PRJNA1465577.
