# Supplementary material for: Arbuscular mycorrhizal associations persist in flooded sediment, with distinct communities in soil-borne and sediment-borne roots of Phragmites australis
Source: Front Fungal Biol. 2026 Jul 15;7:1815819. doi: 10.3389/ffunb.2026.1815819 (PMC13416099; doi:10.3389/ffunb.2026.1815819)
Supplement: Supplementary file 1 [file SupplementaryFile1.docx]

**Supplemental Methods**

**Colonisation metrics**

Mycorrhizal colonisation was assessed by scoring individual root fragments based on their colonisation intensity, arbuscule abundance, and vesicle abundance folowing the Trouvelot method (Trouvelot and Gianinazzi 1986). Each root fragment was scored for colonisation intensity on a scale of 0-5, while arbuscule and vesicle abundance were scored on a scale of 0-3 (Figure S2). These scores were then used to calculate the intensity of colonisation (M%), arbuscule abundance (A%), and vesicle abundance (V%) for each root system, which corresponds to all 15 root fragments assessed per sample. The intensity of mycorrhizal colonization in the root system (M%) was determined according to formula (1). The values 95, 70, 30, and 5 used in Formula 1, and the corresponding values used in Formula 4, are weighting coefficients derived from the established Trouvelot method for estimating arbuscular mycorrhizal colonisation intensity and arbuscule abundance in root systems. Specifically, these coefficients correspond to representative values assigned to the ordinal colonisation classes used in the Trouvelot scoring system. They allow categorical microscopic observations of colonisation intensity to be converted into quantitative estimates of mycorrhizal colonisation. (Trouvelot and Gianinazzi 1986).

$$M\%= \frac{(95n5+70n4+30n3+5n2+n1)}{total number of fragments} (1)$$

Where *n5*, *n4*, *n3*, *n2*, and *n1* represent the number of root fragments assigned colonization scores of 5, 4, 3, 2, and 1, respectively. To derive the arbuscule (A%) and vesicle (V%) abundance, the intensity of colonisation within mycorrhizal root fragments (m%) was first calculated for each root system using the formula (2).

$$m\%=M\% \times\frac{total number of fragments}{number of fragments that are mycorrhizal} (2)$$

Next, arbuscule abundance within mycorrhizal root fragments (a%) was calculated using formulas (3) and (4).

$$a\%= \frac{(100mA3+50mA2+10mA1)}{100} (3)$$

$$mA3=\frac{(95n5A3+50n4A3+30n3A3+5n2A3+n1A3)}{number of fragments that are mycorrhizal} \times\frac{100}{m\%} (4)$$

Where *n5A3n*, *n4A3*, *n3A3*, *n2A3*, and *n1A3* are the numbers of fragments simultaneously assigned colonization scores of 5–1 and arbuscule abundance class A3. Equivalent calculations were performed for A2 and A1. Finally, arbuscule abundance in the entire root system (A%) was determined using formula (5).

$$A\%=a\% \times\frac{M\%}{100} (5)$$

For vesicle abundance in the root system (V%), identical calculations were performed but using assessements of vesicle abundance instead of arbuscule abundance. Lastly, the frequency of mycorrhizal colonization in the root system (F%) was calculated using formula (6).


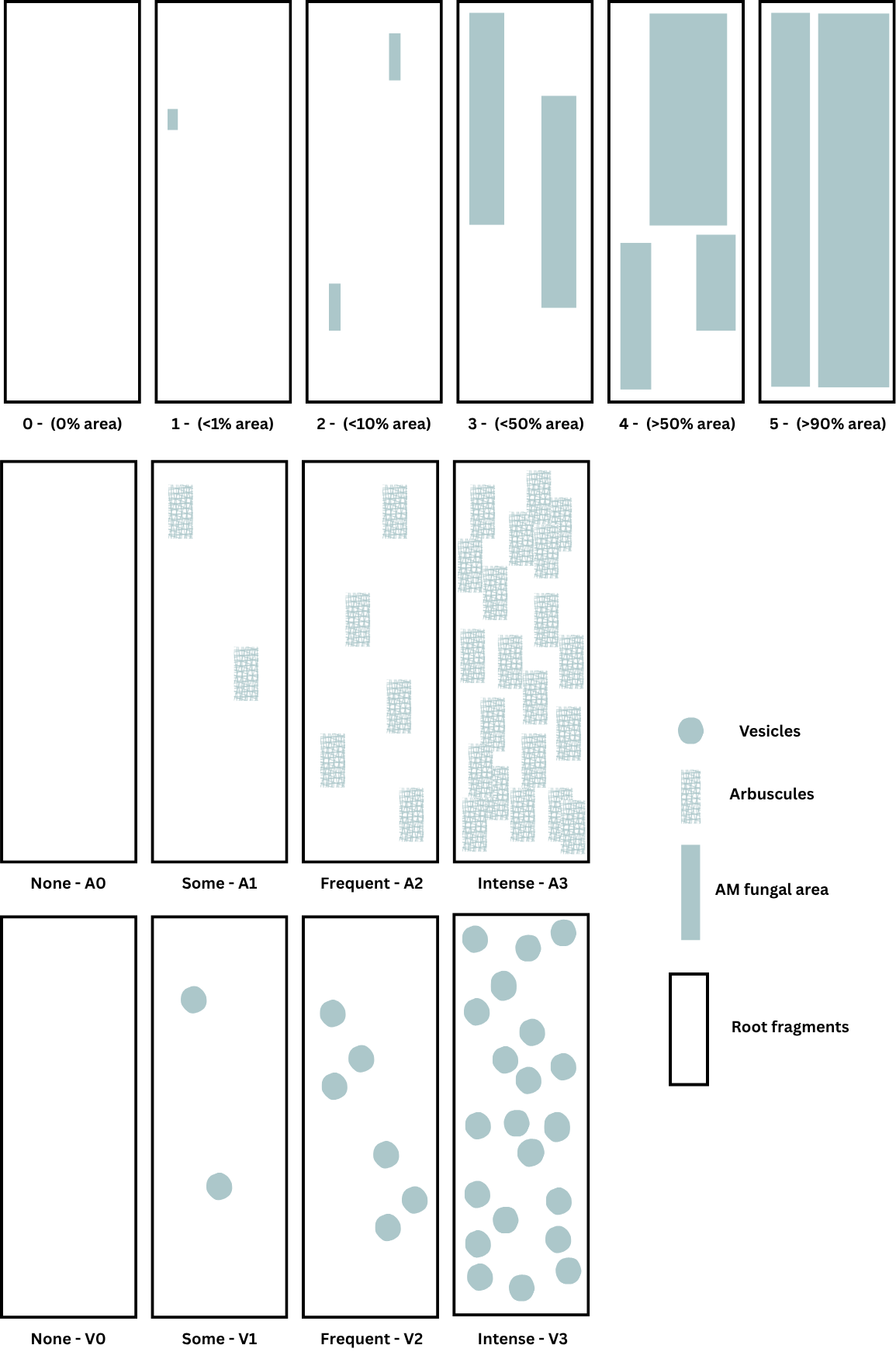


**Figure S1. Illustration of the data that is collected during microscopical AM fungal colonisation assessment.** Colonisation intensity is measured on a scale from 0 to 5, while arbuscule abundance and vesicle abundance is measured on a scale from 0 to 3. Vesicle abundance is not shown in this illustration, but it follows the same concept as arbuscule abundance classification. Scoring method was developed by Trouvelot and Gianinazzi (1986). Illustration created by the authors.


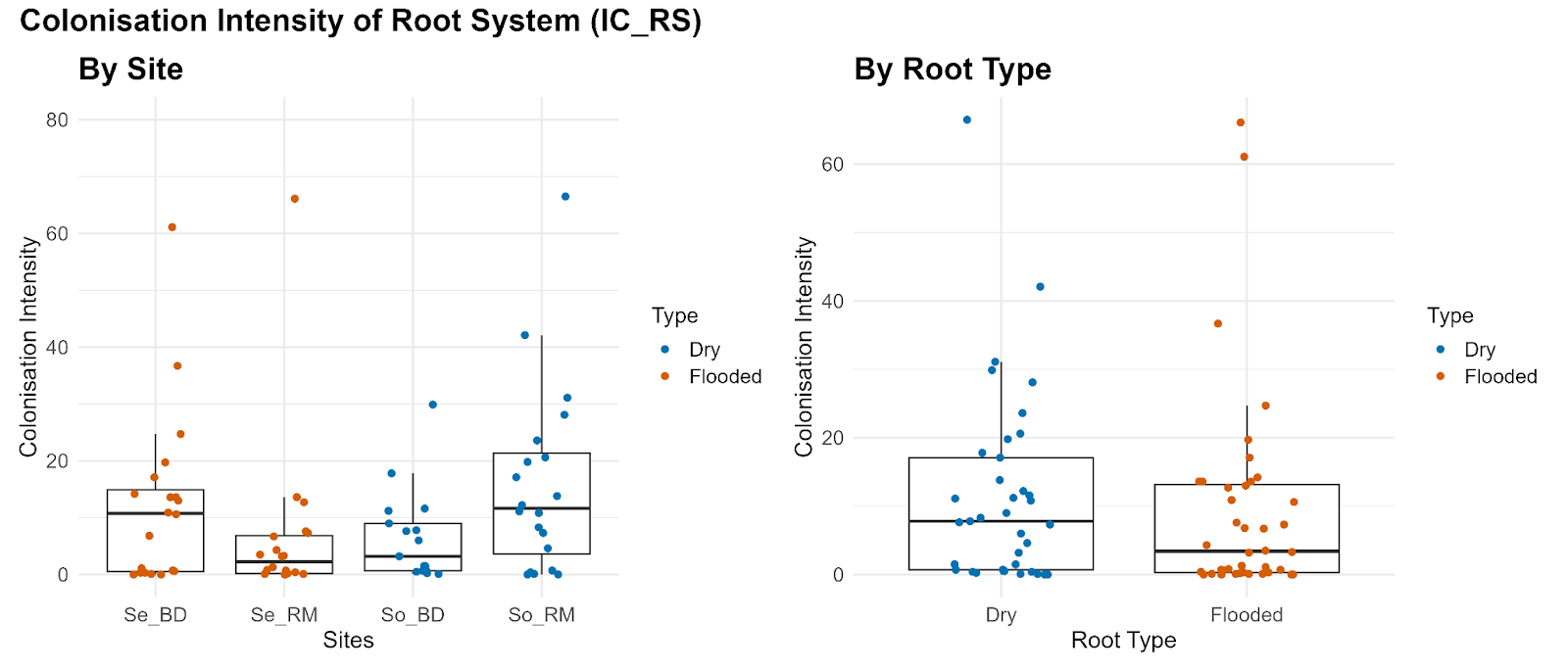


**Figure S2. Intensity of colonisation (M%) within the reed roots.** The intensity of colonisation (M%) was calculated for each sample and is displayed per sampling location and root habitat (A) and for each root habitat across both sampling locations (B)**.** Boxplots show the median as the central bold line, the interquartile range as the lower and upper hinges, and whiskers extending to the 5th and 95th percentiles. Individual points represent biological samples. No significant differences were detected across sites (p= 0.07) or root type (p= 0.09) by Kruskal-Wallis test.

**
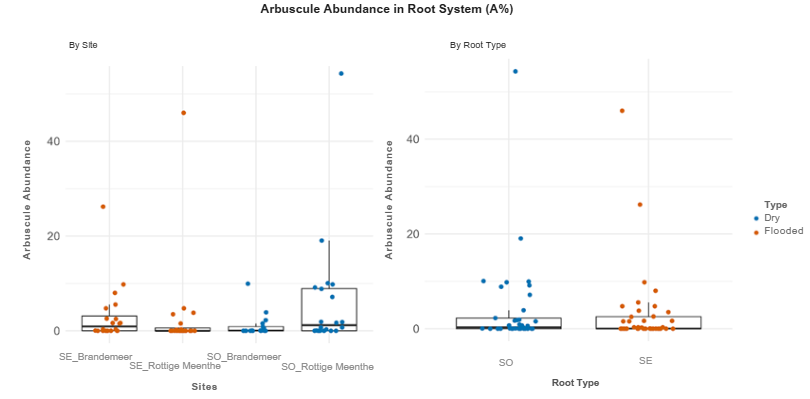
**

**Figure S3. Arbuscular abundance (A%) within the reed roots.** Arbuscule abundance (A%) was calculated for each sample and is displayed per sampling location and root habitat (A) and for each root habitat across both sampling locations (B). Boxplots show the median as the central bold line, the interquartile range as the lower and upper hinges, and whiskers extending to the 5th and 95th percentiles. Individual points represent biological samples. No significant differences were detected across sites (p= 0.08) or root type (p= 0.56) by Kruskal-Wallis test.


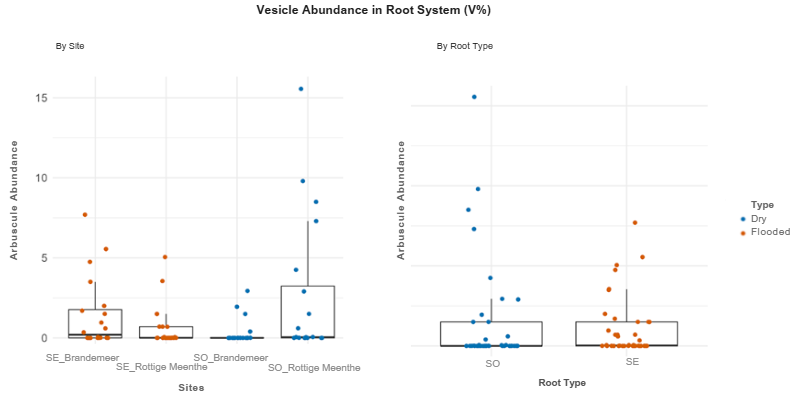


**Figure S4. Vesicle abundance (V%) within the reed roots.** Vesicle abundance (V%) was calculated for each sample and is displayed per sampling location and root habitat (A) and for each root habitat across both sampling locations (B). Boxplots show the median as the central bold line, the interquartile range as the lower and upper hinges, and whiskers extending to the 5th and 95th percentiles. Individual points represent biological samples. No significant differences were detected across sites (p= 0.13) or root type (p= 0.51) by Kruskal-Wallis test.

**
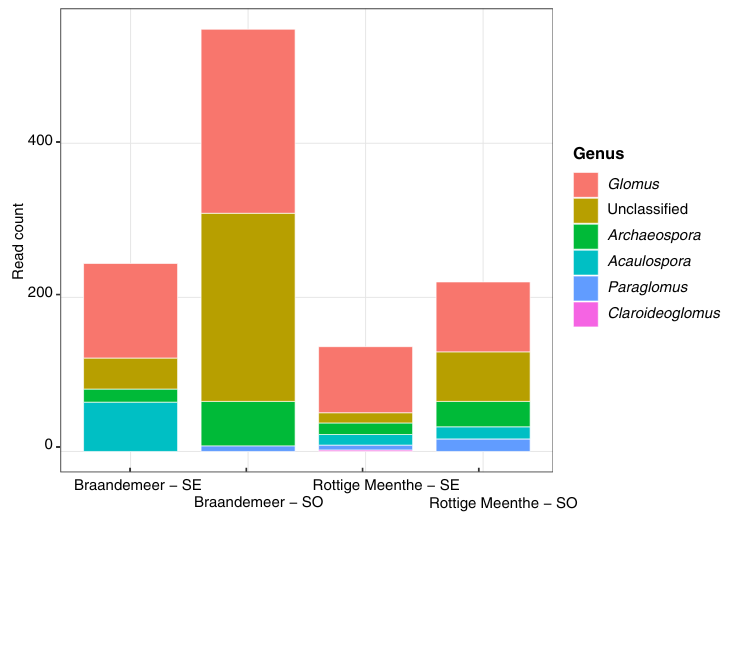
Figure S5. Arbuscular mycorrhizal (AM) fungal read counts by genus across reed root habitats and wetland sites.** Bars represent total AM fungal read counts for each category, with colours indicating genus-level assignments. Samples are grouped by site, Braandemeer and Rottige Meenthe, and by root-associated habitat type: SO, roots collected from soil, and SE, roots collected from flooded sediment.

**Table S1. Evidence for arbuscular mycorrhizal fungi in aquatic and semi-aquatic plants.**

| **Plant species** | **Plant family** | **AM fungal species** | **Reference** |
| --- | --- | --- | --- |
| *Berula erecta* | Apiaceae | *G. claroideum, Scutellospora* sp*.* | (Radhika and Rodrigues, 2007) |
| *Blyxa echinosperma* | Hydrocharitaceae | *G. claroideum, Scutellospora* sp*.* | (Radhika and Rodrigues, 2007) |
| *Canna flaccida* | Cannaceae | *Rhizophagus* sp*.* | [(Calheiros et al., 2019)](https://www.zotero.org/google-docs/?broken=WoqmKn) |
| *Canna generalis* | Cannaceae | *Glomus* sp*.*, *Archaeospora* sp*.* | (Xu et al., 2021) |
| *Canna indica* | Cannaceae | *Rhizophagus* sp*.*, *G. etunicatum* | [(Calheiros et al., 2019)](https://www.zotero.org/google-docs/?broken=WoqmKn) |
| *Centella asiatica* | Apiaceae | *G. claroideum* | (Radhika and Rodrigues, 2007; Xu et al., 2016) |
| *Cyperus alternifolius* | Cyperaceae | *Archaeospora* sp., *Rhizophagus irregularis* | [(Hu et al., 2020; Xu et al., 2021)](https://www.zotero.org/google-docs/?broken=Ob6fo1) |
| *Dorsera indica* | Droseraceae | *G. claroideum* | (Radhika and Rodrigues, 2007) |
| *Eichhornia crassipes* | Pontederiaceae | *Acaulospora* sp., *Archaeospora* sp., *Glomus* sp*.* | (Xu et al., 2016) |
| *Eleocharis palustris* | Cyperaceae | *Endogone* sp*.* | [(Søndergaard and Laegaard, 1977)](https://www.zotero.org/google-docs/?broken=qAjigP) |
| *Eriacaulon cinereum* | Eriocaulaceae | *G. claroideum* | (Radhika and Rodrigues, 2007) |
| *Iris speudacorus* | Iridaceae | *Rhizophagus irregularis* | [(Hu et al., 2020)](https://www.zotero.org/google-docs/?broken=SjHfEH) |
| *Isoetes coromadelina* | Isoetaceae | *G. claroideum* | (Radhika and Rodrigues, 2007) |
| *Isoetes lacustris* | Isoetaceae | *Endogone* sp*.* | [(Søndergaard and Laegaard, 1977)](https://www.zotero.org/google-docs/?broken=qAjigP) |
| *Lindernia ciliata* | Veronicaceae | *G. claroideum* | (Radhika and Rodrigues, 2007) |
| *Littorela uniflora* | Plantaginaceae | *Endogone* sp*.*, *Acaulospora* sp*.*,*Glomus mosseae*, *G. claroideum* | (Søndergaard and Laegaard, 1977; Nielsen et al., 2004) |
| *Lobelia dortmanna* | Campanulaceae | *Endogone* sp., *Acaulospora* sp.,  *Glomus mosseae, G. claroideum* | (Søndergaard and Laegaard, 1977; Nielsen et al., 2004) |
| *Ludwigia parviflora* | Onagraceae | *G. claroideum* | (Radhika and Rodrigues, 2007) |
| *Marsile quadrifolia* | Marsilaceae | *Glomus* sp*.* | (Radhika and Rodrigues, 2007) |
| *Murdania semeteris* | Commelinaceae | *G. claroideum* | (Radhika and Rodrigues, 2007) |
| *Phalaris arundinacea* | Poaceae | *Rhizophagus irregularis* | [(Hu et al., 2020)](https://www.zotero.org/google-docs/?broken=SjHfEH) |
| *Phragmites australis* | Poaceae | *Acaulospora* sp.,*Glomus* sp.*, G. claroideum, G. fasciculatum, Funneliformis mosseae, Archaeospora* sp., *Rhizophagus irregularis, Gigasporaceae* sp., *Paraglomeraceae* sp. | [(Søndergaard and Laegaard, 1977; Wen et al., 2019)](https://www.zotero.org/google-docs/?broken=qAjigP) |
| *Pistia stratiotes* | Araceae | *G. claroideum, Scutellospora* sp*.* | (Radhika and Rodrigues, 2007) |
| *Rotala malampuzhensis* | Lythraceae | *G. claroideum* | (Radhika and Rodrigues, 2007) |
| *Salvinia natans* | Lamiaceae | *G. claroideum* | (Radhika and Rodrigues, 2007) |
| *Watsonia borbonica* | Iridaceae | *Rhizophagus* sp*.* | [(Calheiros et al., 2019)](https://www.zotero.org/google-docs/?broken=WoqmKn) |
| *Lymnophila indica* | Scrophulariaceae | *G. claroideum* | (Radhika and Rodrigues, 2007) |
| *Rotala densiflora* | Lythraceae | *G. claroideum* | (Radhika and Rodrigues, 2007) |
| *Monocoria vaginalis* | Pontederiaceae | *G. claroideum* | (Radhika and Rodrigues, 2007) |
| *Luronium natans* | Alismataceae | *Acaulospora* sp*.* | (Xu et al., 2016) |

**References:**

Calheiros, C. S. C., Pereira, S. I. A., Franco, A. R., and Castro, P. M. L. (2019). Diverse arbuscular mycorrhizal fungi (AMF) communities colonize plants inhabiting a constructed wetland for wastewater treatment. *Water* 11, 1535. doi: 10.3390/w11081535

Hu, S., Chen, Z., Vosátka, M., and Vymazal, J. (2020). Arbuscular mycorrhizal fungi colonization and physiological functions toward wetland plants under different water regimes. *Science of The Total Environment* 716, 137040. doi: 10.1016/j.scitotenv.2020.137040

Nielsen, K. B., Kjøller, R., Olsson, P. A., Schweiger, P. F., Andersen, F. Ø., and Rosendahl, S. (2004). Colonisation and molecular diversity of arbuscular mycorrhizal fungi in the aquatic plants *Littorella uniflora* and *Lobelia dortmanna* in southern Sweden. *Mycological Research* 108, 616–625. doi: 10.1017/S0953756204000073

Radhika, K. P., and Rodrigues, B. F. (2007). Arbuscular Mycorrhizae in association with aquatic and marshy plant species in Goa, India. *Aquatic Botany* 86, 291–294. doi: 10.1016/j.aquabot.2006.10.009

Søndergaard, M., and Laegaard, S. (1977). Vesicular–arbuscular mycorrhiza in some aquatic vascular plants. *Nature* 268, 232–233. doi: 10.1038/268232a0

Trouvelot, A., and Gianinazzi, P. V. (1986). Mesure du taux de mycorhization VA d’un système radiculaire. Recherche de méthodes d’estimation ayant une signification fonctionnelle. *Physiological and Genetical Aspects of Mycorrhizae*, 217–221.

Wen, Z., Li, H., Shen, Q., Tang, X., Xiong, C., Li, H., et al. (2019). Tradeoffs among root morphology, exudation and mycorrhizal symbioses for phosphorus-acquisition strategies of 16 crop species. *New Phytologist* 223, 882–895. doi: 10.1111/nph.15833

Xu, Z., Ban, Y., Jiang, Y., Zhang, X., and Liu, X. (2016). Arbuscular mycorrhizal fungi in wetland habitats and their application in constructed wetland: a review. *Pedosphere* 26, 592–617. doi: 10.1016/S1002-0160(15)60067-4

Xu, Z., Lv, Y., Fang, M., Liu, J., Zeng, H., and Ban, Y. (2021). Diverse and abundant arbuscular mycorrhizal fungi in ecological floating beds used to treat eutrophic water. *Appl Microbiol Biotechnol* 105, 6959–6975. doi: 10.1007/s00253-021-11470-0
